# Bipolar and Laplacian montages are suitable for high-gamma modulation language mapping with stereoelectroencephalography

**DOI:** 10.3389/fneur.2024.1380644

**Published:** 2024-10-16

**Authors:** Takumi Mitsuhashi, Yasushi Iimura, Hiroharu Suzuki, Tetsuya Ueda, Kazuki Nishioka, Kazuki Nomura, Madoka Nakajima, Hidenori Sugano, Akihide Kondo

**Affiliations:** ^1^Department of Neurosurgery, Juntendo University, Tokyo, Japan; ^2^Epilepsy Center, Juntendo University Hospital, Tokyo, Japan

**Keywords:** stereoelectroencephalography, high-gamma modulation, functional brain mapping, epilepsy surgery, electrode montage, auditory naming

## Abstract

**Objective:**

To determine the optimal montage and vocalization conditions for high-gamma language mapping using stereoelectroencephalography.

**Methods:**

We studied 12 epilepsy patients who underwent invasive monitoring with depth electrodes and measurement of auditory-naming related high-gamma modulations. We determined the effects of electrode montage and vocalization conditions of the response on the high-gamma (60–140 Hz) amplitudes.

**Results:**

Compared to common average reference montage, bipolar and Laplacian montages effectively reduced the degree of auditory naming-related signal deflections in the white matter during the stimulus and response phases (mixed model estimate: −21.2 to −85.4%; *p* < 0.001), while maintaining those at the cortical level (−4.4 to +7.8%; *p* = 0.614 to 0.085). They also reduced signal deflections outside the brain parenchyma during the response phase (−90.6 to −91.2%; *p* < 0.001). Covert responses reduced signal deflections outside the brain parenchyma during the response phase (−17.0%; *p* = 0.010).

**Conclusion:**

On depth electrode recording, bipolar and Laplacian montages are suitable for measuring auditory naming-related high-gamma modulations in gray matter. The covert response may highlight the gray matter activity.

**Significance:**

This study helps establish the practical guidelines for high-gamma language mapping using stereoelectroencephalography.

## Highlights

- Optimal settings for measuring auditory naming-related high-gamma modulations were determined using depth electrodes.
- Bipolar and Laplacian montages highlighted the activity of gray matter by reducing extracortical signal deflections.
- Responses to the task without vocalization reduced signal deflection outside the brain parenchyma.

## 1 Introduction

In the surgical treatment of epilepsy, functional brain mapping using intracranial electroencephalography is useful for identifying and preserving eloquent areas (1, 2). Besides the gold standard electrocortical stimulation mapping, high-gamma modulation mapping, functional localization method based on event-related high-gamma activity (>60 Hz), have recently been established (3–5). Event-related increases in high-gamma amplitude are excellent surrogate markers of neural activation (6–10). High-gamma mapping has the benefit that it can be done simultaneously at all electrode sites without the risk of after-discharges associated with electrical stimulation (11). High-gamma mapping using stereoelectrocephalography (SEEG) has been recently studied (12–14). The pros of SEEG are that it can sample deep-seated structures and the bottom of the sulci in addition to the lateral and medial surfaces of the hemispheres (14, 15). The cons are the contamination with far-field potentials or ocular and muscular artifacts (16, 17). A previous study showed that saccadic ocular muscle artifacts contaminate the SEEG of the temporal pole in the vicinity of the lateral extraocular muscle and mimic physiological high-gamma activity, although they do not spread to brain regions far from the muscle (16). Another study demonstrated that volume conduction of gray matter activity could contaminate SEEG recorded in the nearby white matter (17). Thus, minimizing the effects of these artifacts to localize brain function accurately using SEEG is necessary.

Proper selection of electrode montage would be one way to reduce artifacts in high-gamma mapping. A previous SEEG study demonstrated that bipolar montage is optimal for evaluation of cortico-cortical evoked potentials (18). No studies determined a standard electrode montage for high-gamma language mapping using SEEG. Previous studies preferentially used bipolar montage (12, 19, 20) or common average reference montages (21, 22) to analyze task-related high-gamma activity in SEEG. Bipolar montage could eliminate common mode signals (e.g., ocular and muscle artifacts) to adjacent electrode contacts and achieve a high local specificity (16, 23). The risk of bipolar montage is that it reduces meaningful signal changes (24). Previous high-gamma mapping studies with electrocorticography (ECoG) preferentially used the common average reference montage, which is free of contamination such as ocular and muscle artifacts in the reference electrode (25, 26). The risk is that the common average reference montage does not sufficiently reduce signal deflections outside the brain parenchyma or in the white matter in SEEG (18).

Selection of vocalization conditions would be the other way to reduce artifacts. Electromyogram (EMG) activity of facial muscles during overt speaking causes signal deflection in EEG (27). The covert vocalization during language mapping could reduce EMG artifacts. Previous EEG and magnetoencephalography (MEG) studies used covert responses to avoid the influence of muscular activation of orofacial muscles during speech production (28, 29). However, no SEEG study has quantitatively shown that the covert response reduces muscle artifacts compared to the overt response in high-gamma language mapping, independent of the montage setting.

This study aimed to determine the optimal montage and vocalization conditions for high-gamma language mapping using SEEG, i.e., settings those reducing artifacts contaminated in the white matter and outside the brain parenchyma and maintaining task-related physiological activity in the gray matter.

**Figure 1:**
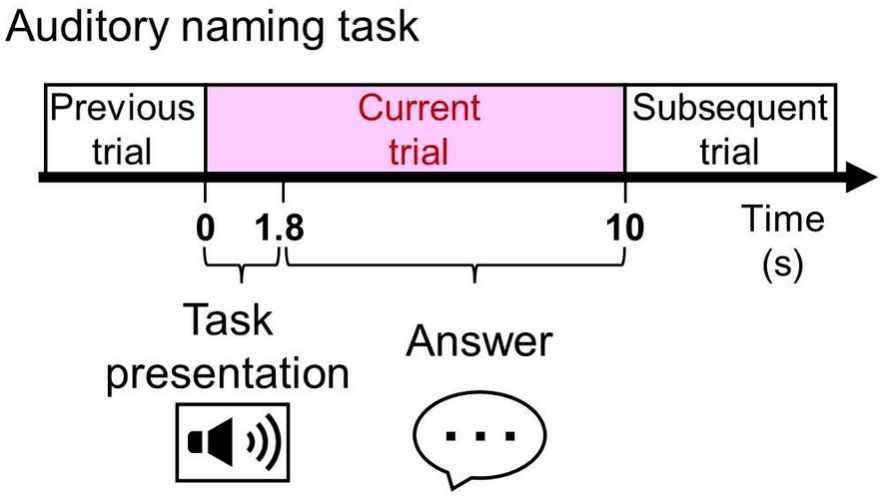
The auditory naming task. Patients were asked to listen to a series of questions, each lasting 1.8 s, and to verbalize a relevant answer overtly or covertly immediately after each question during extraoperative SEEG recording.

[**Aim 1: To determine the optimal montage**] We hypothesized that bipolar and Laplacian montages would reduce far-field potentials and EMG artifacts in electrodes located outside the brain parenchyma and in the white matter, thereby maintaining cortical activity. This hypothesis was motivated by previous SEEG studies that bipolar montage reduced the degree of single-pulse electrical stimulation-related signal deflections at extra-cortical levels, including outside the brain, while maintaining those at the cortical level (18, 30). To address this hypothesis, we studied patients with drug-resistant focal epilepsy who underwent the auditory naming task (Figure 1) with both overt and covert responses during extraoperative SEEG recordings. We calculated and animated task-related high-gamma modulations at 60–140 Hz for each 10-millisecond time window in each montage and vocalization condition. To facilitate understanding, we animated high-gamma modulations during the task for each montage and vocalization condition.

[**Aim 2: To determine the effect of vocalization conditions**] We hypothesized that EMG artifacts would be reduced in the covert response compared to the overt response, resulting in less signal deflection at electrodes located outside the brain parenchyma. This hypothesis was motivated by the notion that MEG and EEG suffer from contamination by the EMG activity of the facial muscles involved in overt speaking (27).

## 2 Methods

### 2.1 Participants

The inclusion criteria included: (a) extraoperative SEEG recording at the epilepsy center of Juntendo University Hospital in Tokyo, Japan between October 2021 and September 2023, (b) functional brain mapping with measurement of language task-related SEEG signal changes as described below. We performed all procedures as part of our routine presurgical evaluation, and the spatial extent and duration of SEEG recording and types of tasks were determined based on clinical demands (25, 31). The Juntendo University Institutional Review Board has approved the present study. We obtained written informed consent from the patients or their legal parents/guardians.

### 2.2 Intracranial electrode placement

All patients had platinum depth electrodes implanted stereotactically using the ROSA robotic trajectory guidance system (Zimmer Biomet, Warsaw, IN, United States). An anchor bolt tightly secured each SEEG depth electrode and minimized movement-related artifacts. Each depth electrode had either six or ten contacts (length: 2.41 and 2.29 mm, respectively; center-to-center distance: 10 and 5 mm, respectively; AD-TECH, Racine, WI, United States).

### 2.3 Imaging process

**Figure 2:**
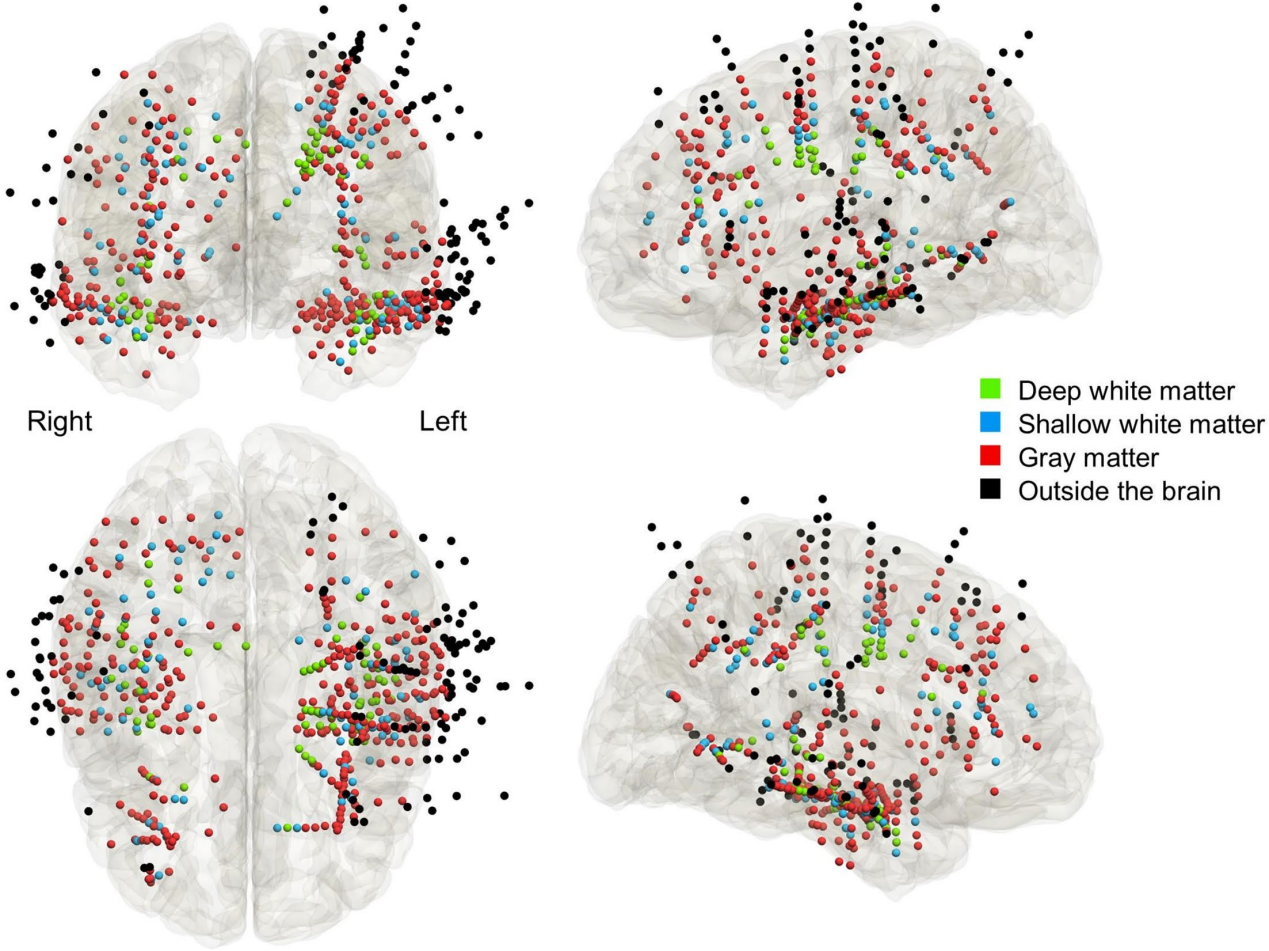
The anatomical locations of depth electrode contacts included in the analysis. Light green: deep white matter. Light blue: shallow white matter. Red: gray matter. Black: outside the brain parenchyma.

Using a pre-implant T1-weighted spoiled gradient-recalled echo sequence MRI and a post-implant CT image, we created a fusion image accurately denoting SEEG depth electrodes within the brain (14, 18, 32). Based on the assessment of coronal, axial, and sagittal fusion images, a given depth contact was classified into one of the following anatomical categories as previously defined (18): (a) deep white matter (white matter deeper than the bottoms of two adjacent sulci), (b) shallow white matter (white matter more superficial than the bottoms of two adjacent sulci), (c) gray matter (i.e., cerebral cortex), (d) cerebrospinal fluid space (i.e., ventricle and subarachnoid space), and (e) outside the arachnoid surface of the brain. We used the Statistical Parametric Mapping 12^1^ to spatially normalize each electrode to the standard Montreal Neurological Institute space [Figure 2; (14, 18, 32)].

### 2.4 Extraoperative SEEG recording

We acquired SEEG data at the bedside with a sampling rate of 1,200 Hz using a g.HIamp biosignal amplifier (g.tec medical engineering GmbH, Austria). The original reference was the voltage of a white matter contact. We excluded electrode sites in the seizure onset zone (25), those located in the cerebrospinal fluid space, and those continuously affected by artifacts from further analysis.

### 2.5 Functional language mapping

We measured language task-related modulations of SEEG signals using a method similar to those previously reported (14, 31, 33). None of the patients had a seizure within 2 h prior to or during task performance. While awake and comfortably seated in a room with unwanted noises minimized, patients received the auditory naming task with vocalized (overt) and non-vocalized (covert) responses (i.e., a total of two tasks: Figure 1). Each task consisted of 100 trials with unduplicated stimuli. We used the Japanese auditory stimuli provided online (31). All stimuli have a 1.8-s duration. Stimuli were presented every 10 s. Patients were instructed to answer “I do not know” in Japanese when they did not know the answer or did not understand a stimulus.

### 2.6 Selection of electrode montage for assessment of language task-related responses

We re-referenced SEEG signals using EEGLAB v2019.1 (34) and analyzed event-related responses using the three different montages. (Common average reference montage) the reference consisted of the average of SEEG signals of all channels. We computed the average reference voltage by excluding all of the SEEG signals at the seizure onset zone, irritative zone, artifactual channels (25), and those outside the brain (18, 35). (Bipolar montage) Each electrode was referenced to the adjacent electrode on a deeper side. (Laplacian montage) Each electrode was referenced to the average of adjacent electrodes on both sides (17, 18, 36).

### 2.7 Measurement and visualization of task-related high-gamma modulations

**Figure 3:**
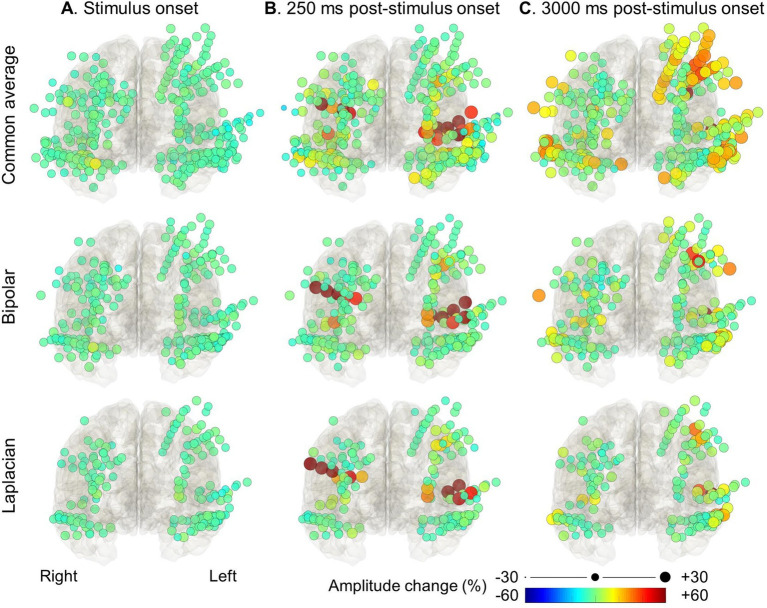
High-gamma modulation during the auditory naming task with overt responses measured on different montages. The snapshots of Supplementary Video S1 demonstrate the percent change in high-gamma amplitude relative to the baseline period in each electrode montage. The color and size of each circle indicate the degree of amplitude change for a given electrode. (A) Stimulus onset. (B) +250 milliseconds post-stimulus onset. (C) +3,000 milliseconds post-stimulus onset (i.e., +1,200 milliseconds post-response onset).

**Figure 4:**
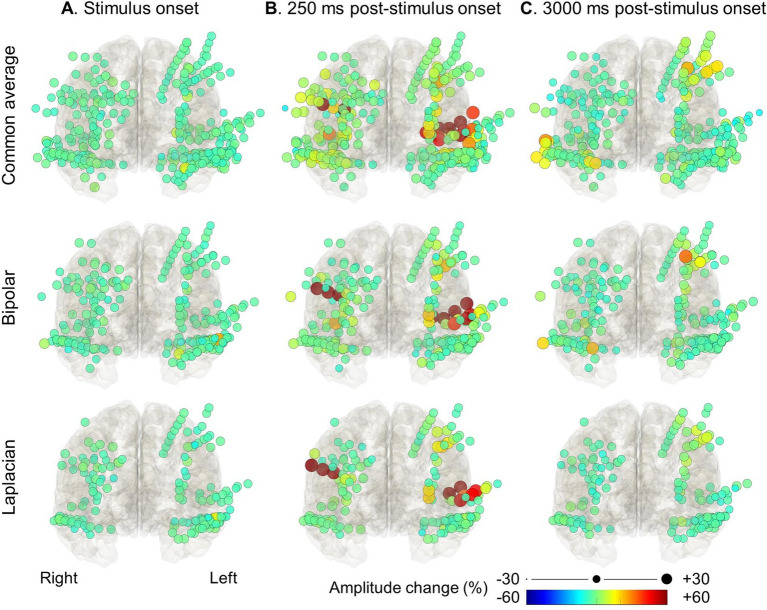
High-gamma modulation during language tasks with covert responses measured on different montages. The snapshots of Supplementary Video S1 demonstrate the percent change in high-gamma amplitude relative to the baseline period in each electrode montage. The color and size of each circle indicate the degree of amplitude change for a given electrode. (A) Stimulus onset. (B) +250 milliseconds post-stimulus onset. (C) +3,000 milliseconds post-stimulus onset (i.e., +1,200 milliseconds post-response onset).

We measured task-related high-gamma activity, as reported in our previous studies (14, 18, 32). We used the FieldTrip toolbox^2^ to perform the Morlet wavelet time-frequency analysis on SEEG signals in the common average reference, bipolar, and Laplacian montages. We transformed SEEG voltage signals from −200 to 5,000 milliseconds period centered on each stimulus onset into time-frequency bins at a 60–140 Hz broadband range [2-Hz frequency bins; a given frequency band divided by seven cycles; sliding in 10-millisecond steps (14);]. Next, we computed the percent change of high-gamma (60–140 Hz) amplitude at each electrode site, compared to the baseline period 200–500 milliseconds prior to stimulus onset for each trial. Finally, we created movies animating the spatiotemporal dynamics of high-gamma amplitude changes in the standard Montreal Neurological Institute brain (Figures 3, 4 and Supplementary Video S1).

### 2.8 Assessment of the effect of electrode montage on task-related high gamma modulations

Mixed model analysis tested the following hypothesis. [1] Electrodes located outside the brain parenchyma and in the white matter would show lower task-related signal changes in the bipolar and Laplacian montages than in the common average reference montage, and electrodes located in the gray matter would maintain signal changes in the bipolar and Laplacian montages. [2] During the response phase, overt responses would elicit higher signal changes outside the brain parenchyma than covert responses. The dependent variable was the maximum high-gamma amplitude change in deep white matter, shallow white matter, gray matter, and outside the brain parenchyma during the stimulus (0–1.8 s) and response (1.81–5 s) phases in a given trial, respectively. The fixed effect predictors included types of electrode montages (common average reference montage, bipolar montage, or Laplacian montage) and vocalization conditions (Overt response or covert response). The random factors included patient and intercept.

### 2.9 Statistical analysis

Statistical analyses were performed using “IBM SPSS Statistics version 27” (IBM Corp., Armonk, NY, United States) and “Statistical and Machine Learning Toolbox MATLAB 2018b” (Mathworks, Natick, MA, United States). The significance was set at an FDR *p*-value of 0.05.

### 2.10 Data and code availability

All SEEG data, as well as the Matlab-based code used in the present study, are available upon request to the corresponding author.

## 3 Results

### 3.1 Configurations and anatomical locations of implanted electrodes

We analyzed the empirical data from 12 patients who underwent high-gamma modulation language mapping during extraoperative SEEG recording (Table 1). SEEG recording was done with 47.8 [±13.6] analyzed depth electrodes per patient on average (standard error). According to the consensus following group discussions, 67 depth electrodes were located at the deep white matter, 86 at the shallow white matter, 309 at the gray matter, and 111 outside the brain (Figure 2).

**Table 1:** Patients profile.

Patient number	Age at surgery (years)	Sex	Sampled hemisphere	Number of included electrodes	Antiseizure medication	Age of seizure onset (years)	SOZ
1	19	Male	Right	24	LCM, PER, VPA, CBZ	12	Rt T, O
2	24	Female	Left	56	LEV	23	Lt T
3	27	Female	Left	64	LEV, LCM	1	Lt F
4	33	Female	Left	60	LCM	12	Lt T
5	35	Male	Bilateral	66	LEV, LTG, VPA, CBZ	14	Blt T
6	10	Female	Right	33	LEV, PER	5	Rt F
7	29	Female	Right	45	LEV, LCM, PER	12	Rt T
8	12	Male	Right	30	LEV, LCM, PER	10	Rt P
9	22	Female	Right	41	LEV, LTG, PER	11	Rt T
10	43	Female	Left	58	LEV, LCM	10	Lt T
11	63	Male	Left	39	LEV, VPA, CBZ	10	Lt P
12	56	Female	Bilateral	57	LCM, VPA, PB	20	Lt T

SOZ, seizure onset zone; LAC, lacosamide; LEV, levetiracetam; LTG, lamotrigine; PER, perampanel; VPA, valpronic acid; CBZ, carbamazepine; ZNS, zonisamide; PB, phenobarbital; F, frontal; p, parietal; T, temporal; O, occipital.

### 3.2 Auditory naming-related high-gamma modulation dynamics on different montages

**Figure 5:**
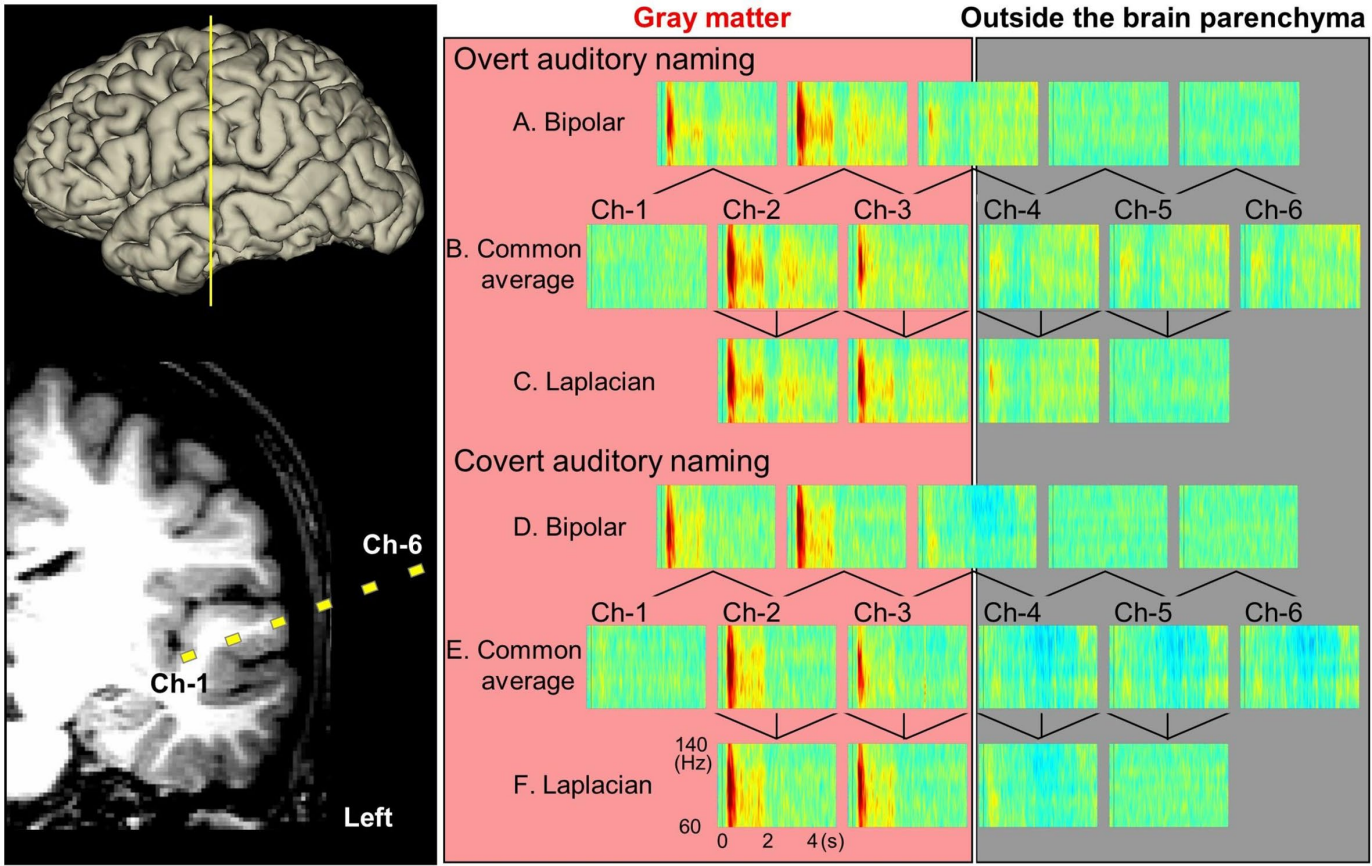
Auditory naming-related high-gamma modulations measured on different montages (patient #2). Yellow squares denote channels 1–6, which recorded task-related high-gamma modulations with overt (A–C) or covert (D,E) responses.

Figures 3, 4 and Supplementary Video S1 demonstrate the spatiotemporal dynamics of high-gamma modulations during the auditory naming task. Electrodes outside the brain parenchyma showed amplitude augmentation in the common average reference montage, especially during overt responses in the response phase (i.e., later than 1,800 milliseconds after the stimulus onset). In bipolar and Laplacian montages, these augmentations decreased, and amplitude augmentation in the adjacent cortex became prominent. Figure 5 shows a representative spectrogram during auditory naming with overt responses. The amplitude changes outside the brain parenchyma seen in the common average reference decreased in the bipolar and Laplacian montages, while those in the gray matter were maintained.

### 3.3 Quantitative assessment of the effect of electrode montage on auditory naming-related high-gamma modulations

**Figure 6:**
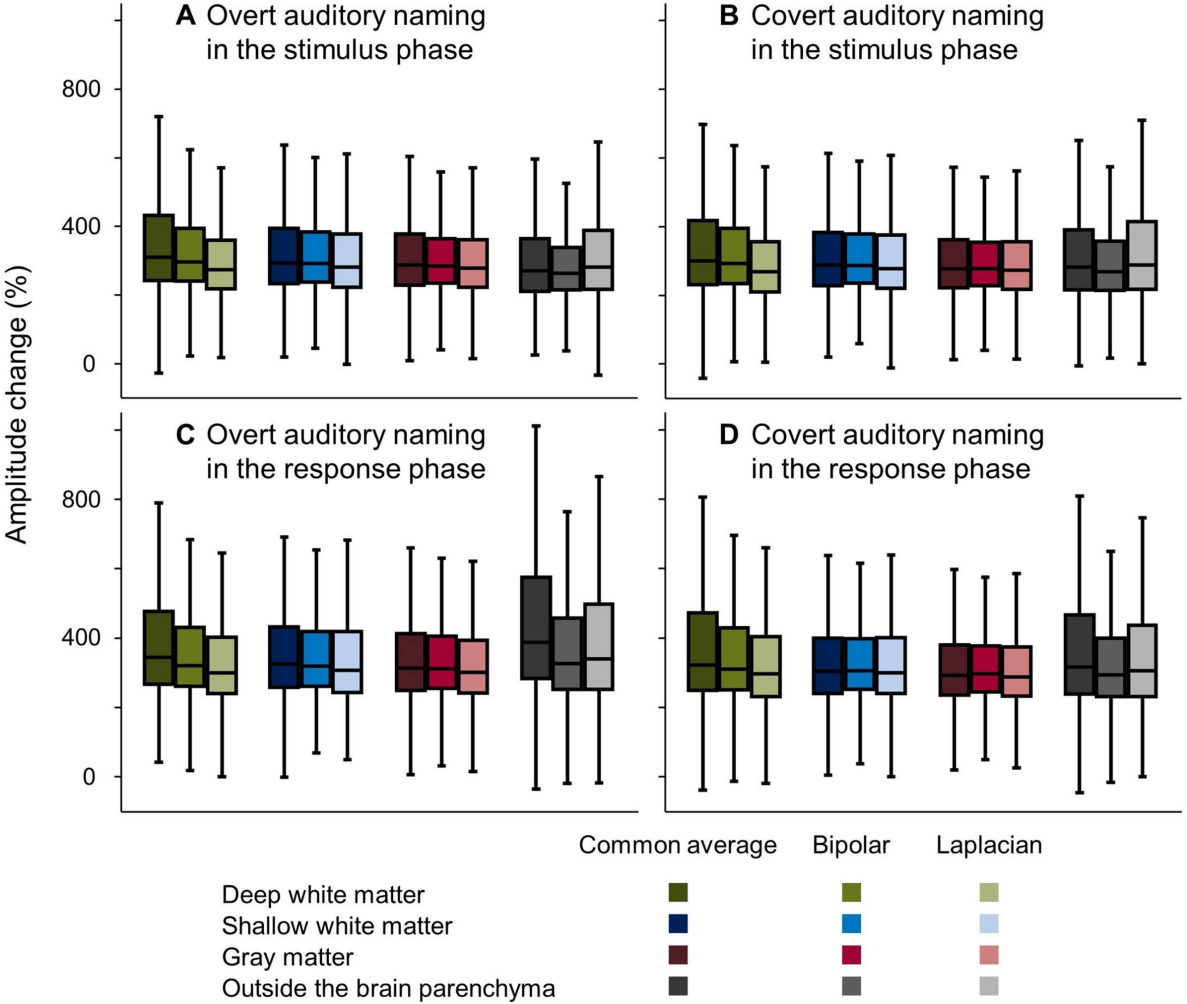
Auditory naming-related high-gamma amplitude changes on different montages. Box plots show amplitude changes on the different montages during the (A,B) stimulus and (C,D) response phases, with vocalized and non-vocalized responses. Here, we present the maximum amplitude changes in the stimulus and response phases, respectively.

Figure 6 presents how well bipolar and Laplacian montages reduced the auditory naming-related high-gamma amplitude recorded at contacts outside the brain parenchyma and in the deep white matter during the response phase. Compared to those measured on common average reference montage, the median amplitude changes during overt naming in the response phase at contacts outside the brain was 62.1% smaller on bipolar montage and 48.8% smaller on Laplacian montage. The median amplitude changes at contacts in the deep white matter were 24.0% smaller on the bipolar montage and 44.6% smaller on the Laplacian montage. Conversely, the high-gamma amplitudes in the gray matter were well maintained on bipolar and Laplacian montages (1.8% smaller on bipolar montage and 11.4% smaller on Laplacian montage). During the stimulus phase, the high-gamma amplitudes in the deep white matter were 12.9% smaller on the bipolar montage and 36.5% smaller on the Laplacian montage. Neither contacts outside the brain parenchyma nor contacts at the grey matter showed more than 10% differences between the montages.

The mixed model analysis (Tables 2, 3) likewise demonstrated that during the response phase, bipolar montage and Laplacian montage reduced the high-gamma amplitudes recorded at contacts outside the brain parenchyma (mixed model estimate: −90.6 to −91.2; *p* < 0.001; *t* = −13.921 to −10.158) and those in the deep white matter (mixed model estimate: −85.4 to −31.5; *p* < 0.001; *t* = −14.827 to −6.254). The high-gamma amplitudes at the gray matter were well maintained on bipolar montage and Laplacian montage (mixed model estimate: +7.3 to +7.8%; *p* = 0.099 to 0.085; *t* = +1.786 to +1.724). During the stimulus phase, bipolar montage and Laplacian montage reduced the high-gamma amplitudes recorded at contacts in the deep white matter (mixed model estimate: −64.5 to −21.2; *p* < 0.001; *t* = −14.652 to −5.499). At the contacts outside the brain parenchyma, the Laplacian montage did not significantly reduce high-gamma amplitudes, and the bipolar montage only reduced mixed-mode estimates by about 20%.

**Table 2:** Mixed model analysis to characterize auditory naming-related high-gamma amplitudes on different montages in the stimulus phase.

Parameters	Estimate	S.E.	df	*t*	Pr(>|t|)	95% CI	
						L.L.	U.L.
Deep white matter							
(Intercept)	+393.5	16.7	33,360	+23.558	<0.001	+360.8	+426.3
CAR (reference)	N/A	N/A	N/A	N/A	N/A	N/A	N/A
BP	−21.2	3.8	33,360	−5.499	**<0.001**	−28.7	−13.6
LAP	−64.5	4.4	33,360	−14.652	**<0.001**	−73.1	−55.8
Covert response	+2.4	3.3	33,360	+0.733	0.464	−4.0	+8.8
Shallow white matter							
(Intercept)	+342.5	11.5	41,378	+29.656	<0.001	+319.9	+365.2
CAR (reference)	N/A	N/A	N/A	N/A	N/A	N/A	N/A
BP	−1.1	2.2	41,378	−0.504	0.614	−5.5	+3.3
LAP	−7.5	2.4	41,378	−3.176	**0.003**	−12.2	−2.9
Covert response	+4.7	1.9	41,378	+2.505	**0.016**	+1.0	+8.5
Gray matter							
(Intercept)	+363.3	13.2	149,882	+27.227	<0.001	+337.3	+389.3
CAR (reference)	N/A	N/A	N/A	N/A	N/A	N/A	N/A
BP	−2.0	3.2	149,882	−0.633	0.614	−8.3	+4.2
LAP	−4.4	3.5	149,882	−1.251	0.281	−11.4	+2.5
Covert response	+12.8	2.8	149,882	+4.644	**<0.001**	+7.4	+18.2
Outside the brain parenchyma							
(Intercept)	+320.7	7.5	45,540	+42.590	<0.001	+305.9	+335.4
CAR (reference)	N/A	N/A	N/A	N/A	N/A	N/A	N/A
BP	−20.1	3.5	45,540	−5.669	**<0.001**	−27.1	−13.2
LAP	−0.8	4.9	45,540	−0.173	0.863	−10.4	+8.7
Covert response	+27.5	3.3	45,540	+8.365	**<0.001**	+21.0	+33.9

S.E., standard error; df, degree of freedom; Pr, probability; CI, confidence interval; LL, lower limit; UL, upper limit. Probabilities for parameters (excluding intercept) with a significance level of 0.05 or less are displayed in bold.

**Table 3:** Mixed model analysis to characterize auditory naming-related high-gamma amplitudes on different montages in the response phase.

Parameters	Estimate	S.E.	df	*t*	Pr(>|t|)	95% CI	
						L.L.	U.L.
Deep white matter							
(Intercept)	+468.2	32.2	33,360	+14.538	<0.001	+405.1	+531.3
CAR (reference)	N/A	N/A	N/A	N/A	N/A	N/A	N/A
BP	−31.5	5.0	33,360	−6.254	**<0.001**	−41.3	−21.6
LAP	−85.4	5.8	33,360	−14.827	**<0.001**	−96.7	−74.1
Covert response	−5.8	4.3	33,360	−1.359	0.232	−14.2	+2.6
Shallow white matter							
(Intercept)	+383.7	14.4	41,378	+26.567	<0.001	+355.4	+412.0
CAR (reference)	N/A	N/A	N/A	N/A	N/A	N/A	N/A
BP	−2.0	2.9	41,378	−0.668	0.504	−7.7	+3.8
LAP	−10.5	3.1	41,378	−3.390	**<0.001**	−16.6	−4.4
Covert response	−15.3	2.5	41,378	−6.170	**<0.001**	−20.2	−10.4
Gray matter							
(Intercept)	+427.0	23.5	149,882	+18.137	<0.001	+380.2	+472.4
CAR (reference)	N/A	N/A	N/A	N/A	N/A	N/A	N/A
BP	+7.3	4.1	149,882	+1.786	0.099	−0.7	+15.3
LAP	+7.8	4.5	149,882	+1.724	0.085	−1.1	+16.7
Covert response	−0.1	3.5	149,882	−0.018	0.986	−7.0	+6.9
Outside the brain parenchyma							
(Intercept)	+518.9	17.4	45,540	+29.810	<0.001	+484.7	+553.0
CAR (reference)	N/A	N/A	N/A	N/A	N/A	N/A	N/A
BP	−90.6	6.5	45,540	−13.921	**<0.001**	−103.4	−77.9
LAP	−91.2	9.0	45,540	−10.158	**<0.001**	−108.8	−73.6
Covert response	−17.0	6.0	45,540	−2.808	**0.010**	−28.8	−5.1

S.E., standard error; df, degree of freedom; Pr, probability; CI, confidence interval; L.L., lower limit; U.L., upper limit. Probabilities for parameters (excluding intercept) with a significance level of 0.05 or less are displayed in bold.

### 3.4 Quantitative assessment of the effect of vocalization conditions on auditory naming-related high-gamma modulations

**Figure 7:**
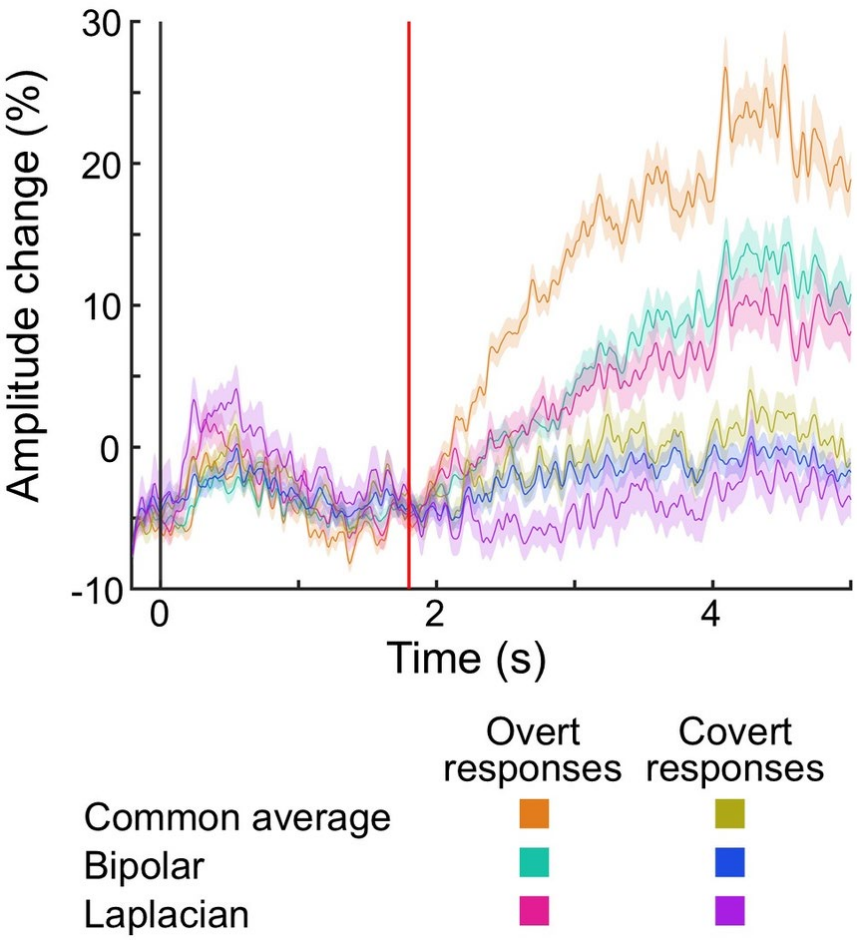
The temporal dynamics of auditory naming-related high-gamma amplitude at electrodes placed outside the brain parenchyma. Plots present the temporal dynamics of high-gamma amplitude on given montages and vocalization conditions. Shading: 95% confidence interval. Black vertical line: stimulus onset. Red vertical line: stimulus offset (i.e., 1.8 s).

Figure 7 shows the changes in auditory naming-related high-gamma amplitudes in each vocalization condition and montage at the contacts outside the brain parenchyma. During the response phase, the overt responses elicited larger amplitude changes than the covert responses. These amplitude changes were smaller in bipolar and Laplacian montages compared to the common average reference montage.

The mixed model analysis (Table 3) demonstrated that overt responses elicited larger amplitude changes outside the brain parenchyma during the response phase than covert responses (mixed model estimate: +17.0; *p* < 0.001; *t* = +2.808). The electrodes in the deep white matter did not show the difference in the high-gamma amplitude between vocalization conditions (mixed model estimate: +5.8; *p* = 0.232; *t* = +1.359).

## 4 Discussion

### 4.1 [Aim 1] Optimal electrode montage

The present study suggests that applying bipolar and Laplacian montages may be optimal for analyzing auditory-naming related high-gamma modulations recorded on SEEG. The present study shows that bipolar and Laplacian montages effectively reduce the signal deflection at contacts in the deep white matter and outside the brain parenchyma, as observed in the common average reference montage, and maintain the high-gamma augmentation in the gray matter. We assumed that in the deep white matter, bipolar and Laplacian montages mainly reduced the far-field potentials. They reduced amplitude changes to the same extent during the stimulus (21.2 and 64.5%, respectively) and response (31.5 and 85.4%, respectively) phases, independent of the presence of speech. A previous SEEG study showed that the white matter signal was mainly a mixture of volume conduction from nearby gray matter (17). SEEG studies analyzing cortico-cortical evoked potentials and spectral responses showed that bipolar and Laplacian montage reduced contaminating far-field potentials in the white matter and outside the brain parenchyma as in the present study (18, 30). We then assume that the reduced signal deflections outside the brain parenchyma mainly consist of EMG artifacts. Bipolar and Laplacian montages reduced amplitude changes by only 20.1 and 0.8% in the stimulation phase and 90.6 and 91.2% in the response phase, respectively. Contacts outside the brain parenchyma also showed a 17% reduction in amplitude change in covert responses. Previous SEEG studies showed the risk of muscle artifacts in the temporal surface region and lateral rectus muscle activity contamination in the temporal pole in the vicinity of the muscle (16, 37).

One may consider that bipolar montage would also reduce significant local activity across two electrodes (24). Nevertheless, the present study showed that bipolar montage reduced gray matter activity in the response phase by only 1.8%, less than deep white matter (24.0%) and outside the brain parenchyma (62.1%).

One may also consider that if MRI and CT can be accurately fused, there is no need to select a montage because electrodes outside the brain parenchyma or in the white matter can be visually excluded. However, the mean cortical thickness is about 2.5 mm, and previous studies reported that MRI was distorted by magnetic field inhomogeneity, resulting in a coordinate variation of about 2 mm compared to CT (38, 39). We believe that the selection of an appropriate montage would complement these image fusion pitfalls and help in the identification of cortical activity.

Our findings support the opinion that bipolar montage is suitable for SEEG interpretation in clinical practice (35, 40). We believe our findings will be useful in developing guidelines for functional brain mapping using high-gamma modulation in SEEG.

### 4.2 [Aim 2] Optimal vocalization condition

We assumed that covert response may be suitable for SEEG mapping in brain regions where the covert response elicits sufficient high-gamma augmentation. The advantage of covert response is that it makes it easier to identify gray matter activity by reducing signal deflection outside the brain parenchyma. In the present study, covert response reduced signal deflections outside the brain parenchyma independent of the montage setting during the response phase. Previous EEG and MEG studies mainly examined covert speech production or pre-vocalization processes to avoid the influence of EMG artifacts associated with vocalization (28). The disadvantage of a covert response is that it may only elicit low amplitude changes and risk missing the language-related areas. Previous ECoG studies showed that the covert response elicited lower high-gamma augmentation in the motor and language areas of the frontal lobe and lower sustained high-gamma augmentation in the sensory language area of the temporal lobe than did the overt response in word repetition tasks (41, 42). Functional areas identified by ECoG high-gamma language mapping using covert responses matched those identified by electrocortical stimulation, excluding the oral motor cortex (43). By exploring the physiological SEEG high-gamma modulation of each brain region for each vocalization condition, further research will determine the optimal vocalization conditions for each brain lobe and region, improving the accuracy of functional localization in the high-gamma language mapping.

### 4.3 Methodological considerations

The present study did not perform simultaneous EMG recordings of muscles for speech production. Jerbi et al. (16) simultaneously recorded electrooculograms and showed the saccade-related gamma band activity in SEEG. Further studies will support our results by showing a direct correlation between artifacts and EMG by simultaneous EMG recordings of muscles involved in speech production, such as the temporal muscle.

This study failed to determine whether bipolar or Laplacian montage is better in high-gamma language mapping. Each electrode montage has the pros and cons (18). Laplacian montage does not allow us to measure neuronal responses at the end of depth electrodes accurately. Bipolar montage may not tell us which pair of contacts is responsible for a given neuronal response of interest.

Most patients in this study did not undergo electrocortical stimulation mapping, making it difficult to determine the accuracy of high-gamma mapping in each montage with respect to electrocortical stimulation mapping. Further studies including patients who underwent both electrocortical stimulation mapping and high-gamma mapping are warranted to provide direct evidence for the superiority of bipolar and Laplacian montages in localizing language areas.

The present study failed to determine the appropriate montage for each language area [i.e., language comprehension-related and language production-related areas (44),] due to the difficulty in determining to which language area the electrodes outside the brain parenchyma belonged. Each language area shows different high-gamma modulation dynamics in the stimulus and response phases (14). Further studies comparing the results of electrocortical stimulation and high-gamma mapping at cortical electrodes in each language area and task phase may help determine the optimal montage and task phase to localize each language area.

## 5 Conclusion

In conclusion, the present study suggests that on depth electrode recording, bipolar and Laplacian montages are suitable for measuring auditory naming-related high-gamma modulations in the gray matter by reducing the extracortical signal deflections. Covert response may also highlight the gray matter activity by reducing EMG artifacts outside the brain parenchyma. Further studies will determine the optimal montage and vocalization condition for high-gamma language mapping for each brain region.

## Data Availability

The raw data supporting the conclusions of this article will be made available by the authors, without undue reservation.

## Footnotes

^1^ http://www.fil.ion.ucl.ac.uk/spm/software/spm12/

^2^ http://www.fieldtriptoolbox.org/
